# Can differences in innovativeness between European cross-border regions be explained by factors impeding cross-border business interaction?

**DOI:** 10.1371/journal.pone.0258591

**Published:** 2021-11-11

**Authors:** Sabine Neuberger, Helmut W. Saatkamp, Alfons G. J. M. Oude Lansink, Dietrich Darr

**Affiliations:** 1 Business Economics Group, Wageningen University & Research, Wageningen, The Netherlands; 2 Faculty of Life Sciences, Rhine-Waal University of Applied Sciences, Kleve, Germany; Szechenyi Istvan University: Szechenyi Istvan Egyetem, HUNGARY

## Abstract

Business interaction is important for innovation performance but may be challenging in cross-border regions. The objective of this research was to investigate the relation between factors that define cross-border business interaction and innovativeness. From the cross-border regional innovation systems literature, we operationalized thirty-five factors which potentially influence cross-border business interaction; these factors concern availability of science and knowledge bases, socio-cultural proximity, accessibility, institutional set-up, and governance. We conducted a survey focusing on these factors and analyzed the data using Cronbach’s alpha and linear regression. The cross-border interaction factors identified in the survey results served as independent variables and the differences in innovativeness levels in different European cross-border regions served as our dependent variable. This study confirmed that differences in innovativeness levels between countries can be related to factors hindering cross-border business interaction.

## Introduction

Despite a common market and the free movement of goods in the European Union (EU), national borders continue to be in place politically and administratively. Forty percent of the EU territory is classified as cross-border regions (the area which touches a 25 km zone to the border), falling on either side of the 40 internal land borders within the EU [1, 2]. As cross-border regions are fragmented by the jurisdiction of two or more different authorities [3], legal and administrative barriers related to European borders reduce the potential economic performance in the border regions by 8.7%, which equals about 3% of the EU’s GDP [4]. Explanations for the lower economic performance of border regions are differences in culture, administrative structures, and infrastructure, which affect business interactions, networking activities, and transportation cost [5]. Such a fragmentation caused by national borders not only has an impact on GDP but also directly affects enterprises’ operations and the efficiency of actors in utilizing local resources [6]. Hence fragmentation through national borders may have more indirect negative effects which are more difficult to measure (e.g. innovation) [7].

The role of innovation as a driver for regional development was already stressed by Lundquist and Trippl [8]. While the level of innovativeness within a country is mostly coherent, the innovativeness levels of neighboring countries are often not on the same level in border regions [9]. van den Broek [10] found that institutional failures in regions with weaker innovation systems are one possible explanation for different levels of innovativeness.

Small- and medium-sized enterprises are driving forces for economic growth, employment creation and innovation development [11]. Enterprises interact with other stakeholders, and establish networks which are context specific and driven by the same goals to effectively utilize resources through, for example, exchange of knowledge [12, 13]. However, networks in cross-border regions are influenced by the national political-administrative structure, the socioeconomic context, geography, and spatial conditions of each country [14]. Hence, cross-border business interactions which we define as interaction (a) between stakeholders (b) from different sides of the border may be hindered by cross-border differences in economic structures, institutional set-ups, and accessibility [8].

Only a few empirical studies have explored the role that a national border plays in business interactions and how innovation can be stimulated and improved in cross-border regions [15]. Makkonen and Williams [16] provided survey metrics and applied them at enterprise level in two Nordic cross-border regions. Peck and Mulvey [17] used a qualitative case study approach to investigate the effect of national borders on the development of an enterprise’s collaboration activities. Both cross-border studies found that changes in the policy debates and economy influence the motivation for business interactions between cross-border countries. The existing literature related to innovation focused on a selection of cross-border regions [18–20] and often used a macro-level perspective [8, 21]. Very little is currently known about the relation of cross-border business interaction and innovation in cross-border regions at a European level.

Hence, the objective of this research is to investigate the relation between factors that define cross-border business interaction and innovativeness in cross-border regions. The cross-border regional innovation system approach served as our conceptual framework to describe and analyze the relationship between level of innovativeness and the factors influencing cross-border business interaction [8, 22]. To advance current knowledge, we conducted a survey and quantitatively analyzed factors for cross-border business interaction in European cross-border regions. This study is the first to use a quantitative survey approach to investigate the relation between regional innovativeness to study cross-border regions at a European level. Furthermore, it provides suggestions for policy makers aimed at facilitating cohesion and economic development across the EU.

## Conceptual framework

Innovation system approaches assume that enterprises can equally benefit and make use of the resources and linkages present within the system [23]. However, particularly in cross-border regions this assumption does not necessarily hold which was the reason for developing a cross-border regional innovation system approach [22]. The cross-border regional innovation system incorporates literature on agglomeration economics [24] and cluster development [25]. Comparable to the differences in proximity [26, 27], the cross-border regional innovation system approach identifies three levels of integration and each level is described by the dimensions, namely nature of linkages, science and knowledge bases, socio-cultural proximity, accessibility (physical proximity), institutional set-up, and governance (economic and policy structure) [8, 22].

### Dimensions of cross-border regional innovation systems and their operationalization in factors

The presence of linkages was emphasized numerous times in innovation literature [23, 28], and therefore we built this research on the premise that cross-border business interaction is a prerequisite for innovativeness in cross-border regions. Linkages are defined as the mere availability of interactions, connections, networks and relationships among stakeholders. Linkages between stakeholders improve the mobilization of resources and the development of knowledge [29, 30]. They are also considered to counteract resistance to change and thereby take an important role for the adoption of innovation and consequently for the economic performance of enterprises [29]. The kind of exchange or flow within a cross-border region can be described as: interactive, symmetrical or asymmetrical, knowledge- or cost-driven [8]. Hence, the interaction between stakeholders at different levels needs continuous reflection on the position in the network and their goals [20].

We derive that linkages, i.e. cross-border business interaction, is the base dimension of the framework and can be hampered by weak science and knowledge bases, a lack of socio-cultural proximity or physical accessibility (infrastructure), unfavorable institutional set-ups, economic structure and policy structures that constrain innovativeness [see e.g. 16, 31, 32]. These other dimensions are described in detail below and Table 1 presents an overview of the dimensions and their operationalization using 35 factors influencing cross-border business interaction.

**Table 1:** Operationalization of dimensions in factors influencing cross-border business interaction.

Theory-based dimension	No. of factors	Factors
*Science and knowledge bases*	5	Educational institutions [22, 29, 30]
		Research institutions [22]
		Projects [33]
		Networking events [29, 33]
		R&D funds [33]
*Socio-cultural proximity*	7	Language [21]
		Addressing people [29, 34]
		Hierarchal structures [29, 34]
		Attitudes in doing business: reliability, mistrust [33]
		Prejudice and mistrust [33]
		Working schedules [34] (company internal factors)
		Communication tools [34, 35]
*Accessibility (physical proximity)*	11	Presence of natural barriers [30]
		Travel distance [30]
		Transport infrastructure (highway, train, ships) [30]
		• presence
		• usage
		• quality: efficiency highway infrastructure
		• quality: efficiency train infrastructure
		• quality: efficiency shipping infrastructure
		• quality: physical condition
		• density (traffic jams)
		Internet connection [30, 35]
		Communication costs [35]
*Institutional set-up*	4	Legal system and requirements [33]
		Interaction and cooperation facilitating organization [33]
		Communication among institutions [22, 29, 30]
		“Help desk” abroad [29]
*Governance (economic and policy structure)*	8	Economic situation [29, 35]
		Living standard and purchasing power [21]
		Industrial specialization [22, 29]
		Enterprise specific foci [29]
		Enterprise demands [29]
		Qualified employees [33, 35]
		Government agenda [22, 33]
		Government mistrust [29]

These factors were derived from the literature and only contains factors which already provided positive results. We excluded those factors that did not prove influential in previous research.

Science and knowledge bases encompass the presence of educational and research facilities, research funds, research projects, and workshops and conferences [8, 22]. Attending workshops and conferences offers interesting possibilities for enterprises to develop and apply knowledge and to establish a network [29, 30, 33]. Hence, education and research facilities with their related activities can be considered as facilitators of not only innovation development but also cross-border linkages between enterprises (or industry) and research.

Socio-cultural proximity captures norms, values, and cultures [33] and can be observed in differing hierarchal structures, habits of addressing each other, or attitudes in doing business. Prejudice, general mistrust or both among citizens of cross-border regions influences the willingness and quality of cross-border business interaction.

Accessibility (or physical proximity) of cross-border regions is defined as the presence of natural barriers and the condition of infrastructure [8, 30]. Natural barriers include mountains, rivers, or sea, and can present limitations to direct cross-border business interaction. This limitation is especially severe if transport and communication infrastructure is not sufficiently available in border regions.

Institutional set-up is defined as the degree of similarity in laws and regulations as well as the degree of accordance in plans and goals for future economic progress [8, 22]. Because of country specific differences in the institutional set-up, it is important that organizational infrastructure (such as network organizations, information brokers, other information channels) is present in cross-border regions to provide information on matters in the other countries for e.g. enterprises, but also to link national tasks with tasks of the neighboring regions and countries.

Governance concentrates on the economic and policy structure of cross-border regions. Economic structure refers to the industry specialization and strategies for coherent industry development [8, 22] and can be described for example through the presence of regional competences [22] or unit labor cost [35]. Policy structure of cross-border regions is defined by the political system (centralist versus federalist), modes of operation and governance structures. These can differ among countries in terms of ruling and agenda setting by the nation, or a regional authority such as a province or a city. The availability of instruments of cross-border policy and innovation policy affect enterprises’ innovation activities [8, 22, 33]. Differences, synergies and complementarities of the countries in a cross-border region affect an enterprise’s willingness to cooperate [17].

The underlying assumption is that factors hindering cross-border business interaction might explain differences in innovativeness levels. Hence, we expect that regional innovativeness levels differs in cross-border regions compared to central regions, if obstacles for cross-border business interaction exist. Such obstacles may occur with regard to availability of science and knowledge bases, socio-cultural proximity, accessibility, institutional set-up, and governance (economic and policy structure). The operationalized factors serve as the basis to study cross-border business interaction.

## Material and methods

Two different data sources were used for this research. We collected primary data on cross-border business interaction through an online survey, and secondary data on differences in innovativeness was derived from an indexed measure provided by the European Commission. Below, we first describe the design and implementation of the online survey and second explain the extraction of secondary data.

### Primary data

We used a survey approach because secondary data proxies are barely available at a cross-border level. The questionnaire was based on the conceptual framework and focused on how the dimension *linkages*, i.e. cross-border business interaction, is influenced by the other five dimensions: (a) *science and knowledge bases*, (b) *socio-cultural proximity*, (c) *accessibility*, (d) *institutional set-up*, and (e) *governance*. These five dimensions were addressed in five blocks of questions, each consisting of a closed and an open question and arranged similarly. The closed questions asked whether “Cross-border business interaction is hampered by” any of the 35 factors operationalized in Table 1. The responses were ranked on a 5-point Likert scale (“Not at all”, “Slightly”, “Moderately”, “Very”, and “Extremely”). The open questions provided the option of naming positive or negative examples.

Respondents: In EU regions, regional institutions play an important role in shaping economic growth [36] and cross-border institutions are important for facilitating cross-border cooperation [37]. Therefore, representatives of European cross-border institutions such as Euregio offices were considered as the target group of the survey. We aimed to overcome potential limitations of the study, such as the selection of factors and the total number of questions, by pre-testing with three professionals of the Euregio office Rhine-Waal. The final draft of the questionnaire was finalized with minor adjustments.

The Social Sciences Ethics Committee of Wageningen University & Research retrospectively approved this study. When the study began in 2018, Wageningen University rules did not require to obtain explicit consent for surveys and therefore, we did not include a separate section in the survey. However, we had fully informed participants about the aim of the study, use of results, and that all data was processed anonymously. Therefore, we conclude that everything was done to fulfill the anonymity and information requirements to the participants.

The survey was available online from September 18 through October 31, 2018. For reasons of user-friendliness, the survey was conducted online using the provider “Sosci-Survey” (www.soscisurvey.de) because of the provider’s location in Germany and its liability to the German law of data security. Access was provided by a link sent by email to cross-border region institutions. In total, 96 institutions were contacted. Two reminders were sent after 10 and 18 days while the survey was available online. The overall response to the survey was 26%. Due to the low response rate and the design of the survey, our research was limited to exploring whether a relationship of the factors influencing cross-border business interaction and differences in levels of innovativeness exists, but it was not possible to investigate the causality of the relationships between the factors and innovativeness.

### Use of secondary data

We collected secondary data on regional innovativeness from the “Regional Innovation Scoreboard” (RIS) [9]. The RIS is established by the European Commission as a tool to assess and to compare the innovation performance of innovation systems in European regions and is measured at two different NUTS levels, i.e. the ’Nomenclature of territorial units for statistics’. The EU introduced NUTS to divide the economic territory of the EU for conducting regional statistical and socio-economic analysis of the regions, and framing of EU regional policies. It consists of 4 levels, whereas NUTS 0 is country level (e.g. Germany) and NUTS 3 is the smallest diversification for specific diagnoses of regions [38]. The RIS is available at NUTS 1 and NUTS 2 level. It is an indexed measure based on 16 indicators, such as population with tertiary education, scientific co-publication, and R&D expenditure in the public sector and the business sector [9]. We consider different RIS levels on either side of the border as underutilized innovation potential.

One limitation of RIS is that data relating to each of the 16 indicators used for RIS index calculation in each region is not always available, resulting in differences in inputs for RIS index calculation between countries. Furthermore, RIS measures tend to measure research driven innovations, and they do not include regional specialization [7]. Despite these shortcomings of RIS, we decided to use this score because (1) it was emphasized in the literature that not one single measure can account for the level of innovativeness, (2) individual countries measure innovativeness differently, and there is no other database available to compare regions on a European level, and (3) RIS is used by the EU to develop policy action plans [see also 7].

A second limitation of the RIS is that it provides information at **national level of NUTS regions** and therefore never addresses a cross-border region. Cross-border regions are defined as the area of all NUTS 3 regions within 25 km from the border, also if the NUTS 3 region is only partially located in that zone [2]. The RIS data is only available at the NUTS 2 and sometimes even only at the NUTS 1 region level. Thereby, it does not coincide with the EU definition of cross-border regions based on NUTS 3 level. Because our survey addressed the level of **cross-border regions**, we calculated the difference in innovativeness levels between the countries of a cross-border region (RIS_diff_) and thereby addressed the problem of different national levels of observation. Occasionally, a cross-border region also covered an area of several NUTS regions within one country. In such cases, the in-country mean among the according regions was calculated first before calculating the difference in RIS. An illustration of RIS_diff_ calculation can also be found in the S1 File.

### Internal consistency of questionnaire design

We tested the factors that were derived from the conceptual framework (Table 1) on their ability to coherently describe one dimension, i.e. internal consistency. Standardized Cronbach’s alpha (*α_st_*) was used to calculate internal consistency of the factors within each dimension:

$$\alpha_{st}=\frac{n\bar{r}}{1+(n-1)\overset{=}{r}},$$ (Eq 1)

where *n* is the number of factors and $\bar{r}$ the average correlation between the factors using Kendall’s tau correlation coefficient. Instead of the Pearson correlation coefficient, it was considered appropriate to calculate the correlation coefficient based on Kendall’s tau because our dataset contained non-parametric data with ordinal scale measures. Kendall’s tau measures the degree of association between two variables, i.e. factors, without carrying any assumption about the distribution of data. Compared to Spearman’s rho, Kendall’s tau has usually smaller values, is insensitive to error, and is more accurate with smaller sample sizes [39].

Internal consistency is indicated by Cronbach’s Alpha being above 0.7 [40, 41]. Table 2 presents the exact values for Standardized Cronbach’s Alpha and 95% confidence interval. Results show that internal consistency was provided in all dimensions which means that the operationalized factors of the five dimensions were coherently addressed through the variables that were measured in the questionnaire.

**Table 2:** Standardized Cronbach’s Alpha for each dimension.

Dimension	No. of factors	Standardized Cronbach’s Alpha	Lower CI	Upper CI
Science and knowledge bases	5	0.827	0.72	0.94
Socio-cultural proximity	7	0.718	0.55	0.88
Accessibility	11	0.867	0.79	0.94
Institutional set-up	4	0.785	0.65	0.92
Governance	8	0.774	0.64	0.91

### Exploring relationships

The analysis included two steps. First, we conducted a linear regression analysis: our dependent variable is the difference of RIS in a cross-border region (RIS_diff_), while the factors addressed in the survey serve as our independent variables. Four observations were excluded from the analysis due to missing data about regional innovativeness in Russian regions, resulting in n = 23. Second, we investigated whether region specific differences exist between the factors and levels of innovativeness. Compared to the first analysis step, where each factor was considered individually in the regression analysis, we used the dimension’s mean (across all factors of one dimension) in the second analysis step to identify region specific differences. In that sense, we followed Makkonen and William’s [16] suggestion to use the mean if the internal consistency shown in Cronbach’s alpha was high.

To identify region specific differences, we decided on three subsets of regions and sequentially excluded them from the analysis. The first subset consisted of Slovakia, Czech Republic, Hungary, Poland, and Lithuania, i.e. the EU member states that entered the EU with the 2004 enlargement because results could be biased due to their late entry to the EU that limited their time to catch up or adjust to other EU countries. The second subset consists of non-EU countries, i.e. Russia, Switzerland, Liechtenstein, and Norway because of a lack in strong EU policies and support instruments. The third subset includes only Germany because it is overrepresented in the results with 50% of the survey responses in cross-border regions encompassing Germany.

## Results

### Cross-border regions covered

**Fig 1:**
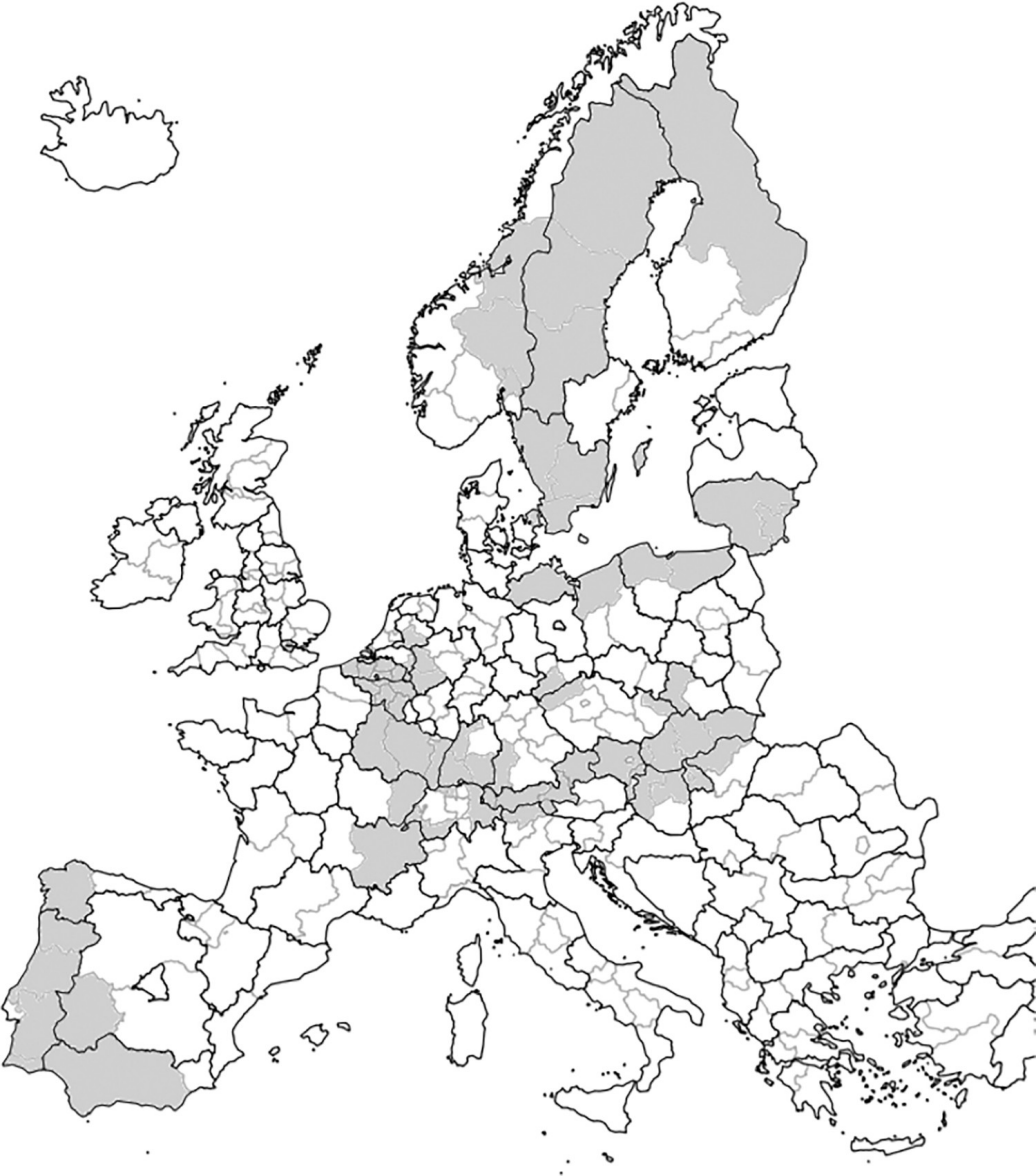
Map of cross-border regions included in the study, presented at NUTS 1 and 2 level. (Source: Modified after Eurostat 2021; © EuroGeographics for the administrative boundaries). The 17 EU countries included Austria, Belgium, Czech Republic, Denmark, Finland, France, Germany, Hungary, Italy, Liechtenstein, Lithuania, the Netherlands, Poland, Portugal, Slovakia, Spain, and Sweden (alphabetical order). Three of the countries were non-EU countries, namely Russia, Switzerland, and Norway.

Survey responses were obtained for 17 different European cross-border regions and corresponded to 20 different countries. Fig 1 shows the regions from which data were obtained.

Survey respondents stem from cross-border regions with different levels of innovativeness, covering a mean RIS between 61 and 140, where 100 indicates the European mean [9]. The difference of innovativeness levels (RIS_diff_), i.e. the difference among different countries of a cross-border region, ranged between 6 and 111 (see S1 Table).

### Exploring the relationship between factors defining cross-border business interaction and innovativeness

Regarding the first analysis step which included all regions, we observed that factors hindering cross-border business interaction are stronger in cross-border regions with a large RIS_diff_ than in border regions with a small RIS_diff_. Such a positive relationship was found in 33 out of 35 factors (94%) on RIS based on our linear regression model which is in line with our expectation. However, three factors showed a negative relationship and are not in line with our assumption. The respective factors were: different habits of addressing people (e.g. greeting, first or last name), differing hierarchal structures in businesses, and differing approaches and attitudes in doing business (see S1 Fig).

In the second analysis, we excluded subsets to investigate whether specific differences in the regions exist between the relationship of factors and innovativeness levels. Similar to the first analysis, we found positive relationships, i.e. ascending slopes of the dimension’s mean: solid line) in four of the five dimensions. In the dimension *socio-cultural proximity*, the subset “excluding non-EU countries” acted against our expectations by showing a descending slope. Hence, we also observed varying subset-specific differences.

**Fig 2:**
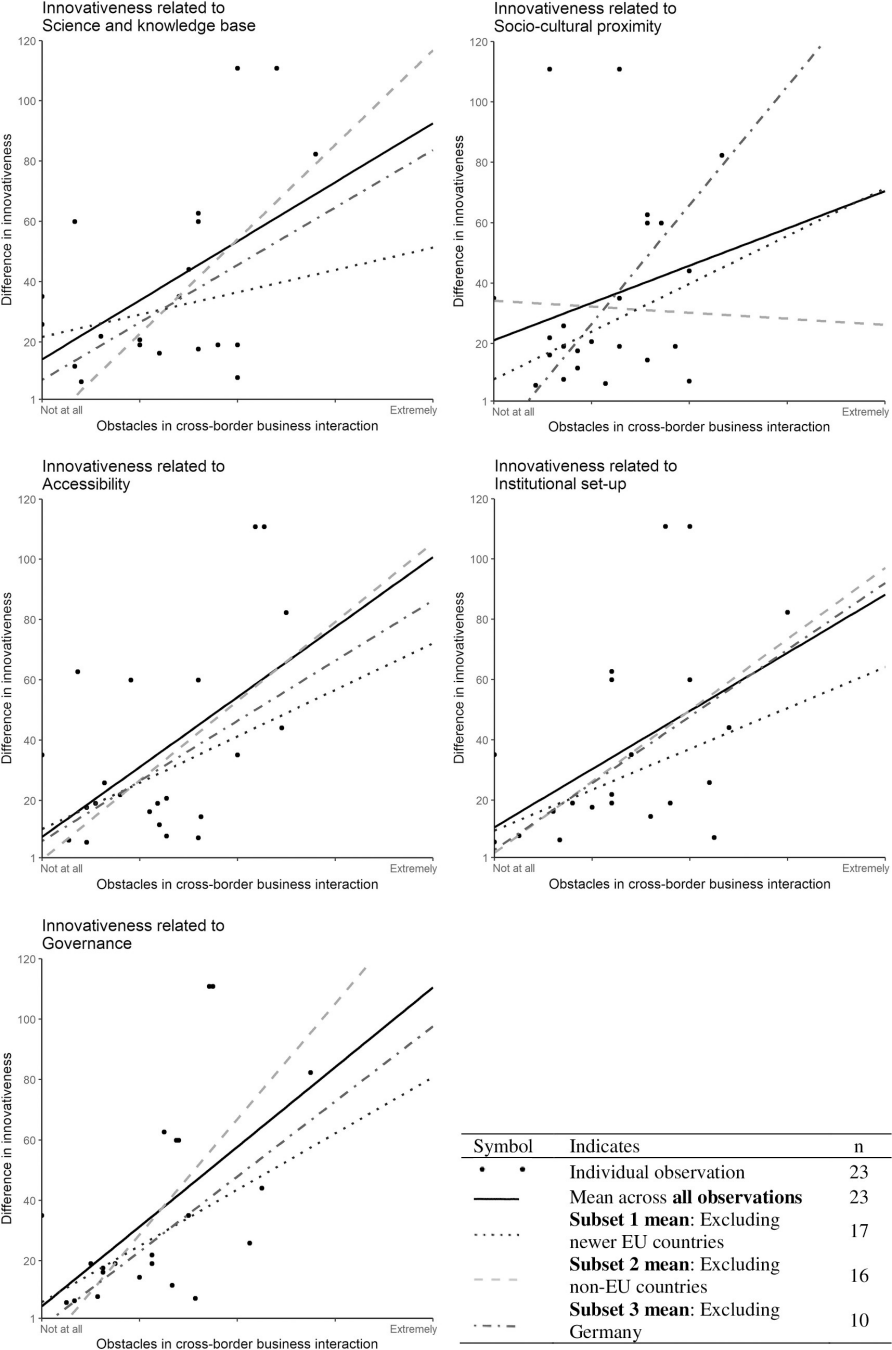
Region specific differences in innovativeness. Source: Own elaboration based on survey data and RIS **[9]**.

The results are illustrated in five graphs, one for each dimension (Fig 2). In all graphs, the y-axis refers to the difference of regional innovativeness levels measured from 1 to 120: “1” indicating a low and “120” indicating a high level of inequality on the level of innovativeness. The x-axis represents the mean of each dimension, and the boundaries are defined by the 5-point Likert scale used in the survey, where the lowest score, i.e. “not at all hampered” can be found on the left side, and the highest, i.e. “extremely hampered” on the right side of the x-axis. The black dots refer to the results of all observations (n = 23) and the fitted lines illustrate the relationship between each dimension and the level of innovativeness. The solid line refers to all observations, while the three additional lines in the scatterplots present the three subsets (i.e. newer EU countries, non-EU countries, and Germany) and show the results excluding the selected cases.

## Discussion

In this study, we focused on the question whether differences in innovativeness levels within European cross-border regions can be explained by factors that influence cross-border business interaction. To answer this question, a conceptual framework was developed based on the cross-border regional innovation system approach. The framework emphasizes the importance of linkages or interactions among enterprises and external stakeholders such as governmental institutions or education and research facilities. We operationalized factors defining cross-border business interaction from the cross-border regional innovation system dimensions and considered linkages, i.e. cross-border business interaction, as the base dimension assembling the other dimensions, i.e. science and knowledge bases, socio-cultural proximity, accessibility, institutional set-up, and governance. The factors were investigated through a survey since empirical studies specifically devoted to these cross-border regions are scarce.

In the remainder of this section, first, we discuss each dimension and use open question responses from our survey to provide specific examples of topics which influenced cross-border business interaction (these will be highlighted in *italics*). Second, we present implications for future research and policy making.

### The relationship between the dimensions and difference in innovativeness

Each cross-border region has its own local peculiarities [42], hence the balance of the five dimensions is different in every cross-border region. Our study showed a mainly **positive** relationship between the dimensions and regional innovativeness levels and also identified subset-specific differences: the more factors hindering cross-border business interaction, the greater the difference of the regional innovativeness level between countries. These results indicate that our findings are generally in line with previous research which will be discussed in detail below.

Our study showed that if cross-border business interaction is impeded by the dimension **access to science and knowledge bases,** it leads to increasing difference in the level of innovativeness. This finding confirms the insights of Schäffler *et al*. [43] who revealed that a well-educated labor supply was important for cooperation in the German-Czech border region–even more important than lower wages. One explanation of this positive relationship could be the multi-presence and intermediary role of specific universities in cross-border network structures [14] because collaboration with public research organizations encourages innovation behaviors among employees and the emergence of new ideas that challenge the organizational situation of enterprises [44]. In our survey, one response indicated *a striking balance in science and knowledge bases between regions*, but yet others pointed out that *specialized Federal Research Institutions are partly less accessible for businesses in neighboring countries* and that there is *a lack of public funding*. We derive that less accessible or lacking public research funds results in forming poor conditions for collaboration and learning [see also 33].

Our observation of the positive relationship between **socio-cultural proximity** and difference in the level of innovativeness confirms the results of a qualitative study conducted with Czech-German enterprises in border regions [5]. Leick [5] identified three reasons responsible for different developments of border regions (especially eastern European border regions), among which cross-cultural differences in cross-border business interactions were identified. Balogh and Pete [45] found that a local cross-border culture including language and ethnicity was a significant element for cross-border integration. A real-life situation showing the importance of socio-cultural proximity was provided by one respondent who described a problem of differing business attitudes: *A German director had no trust in an easy going Dutch director who talked about personal issues*, *such as bringing his child to childcare*.

Our results showed a positive relationship of **institutional set-up** and innovativeness levels. This finding reflects those of van den Broek and Smulders [31] who found that the institutional embeddedness of actors influence cross-border regional innovation systems. Respondents considered *Interreg projects* (3 respondents) and *a connection among governmental institutions* (2 respondents) as a facilitator for the establishment of cross-border partnerships, while, on the other hand, *a lack of a common strategy of economic institutions*, *a lack of responsible persons*, *and unclear procedures* constrained cross-border business interaction.

Our study showed a positive relationship between **accessibility** and the difference in levels of innovativeness; responses indicated that the transportation infrastructure in cross-border regions should be improved. Schäffler *et al*. [43] found that regional connectedness is important in cross-border regions, and an improved infrastructure can increase foreign direct investment in cross-border regions. Respondents asked for *establishing a rail connection* (10 respondents) and *public transportation* (4 respondents), and were concerned with the *quality of (highway) roads* (4 respondents) and the *(re-)construction of bridges* (3 respondents). To give an example of the importance of a well-established infrastructure: the mutual willingness to reconstruct bridges along the Slovak-Hungarian border turned out to be a crucial step for cross-border flows [45]. Concerning communication infrastructure, the *establishment of high-speed internet* in rural areas is a problem specifically affecting cross-border regions (3 respondents). *Roaming costs* were abolished in the EU but still represent a financial burden for non-EU countries (2 respondents). Research on this topic is currently missing, but given the increasing importance of communication infrastructure, it should also be considered in future cross-border studies.

We also found a positive relationship between **governance** (economic and policy structure) and difference in the innovativeness level. According to our respondents, the *mutual acceptance of business qualification* (1 respondent) and *the lack of skilled employees* (2 respondents) increased cross-border business interaction, while *legal obstacles* (1 respondent) or *different technical standards* (1 respondent) made cross-border business interaction difficult. Leick [46] also identified employee recruitment as a motivation for cross-border business interaction. However, motivations can shift in response to changed economic conditions and policy priorities [17], and cross-border business interaction of enterprises also depends on the size and industrial focus of the neighboring market which is also an indication of the importance of the economic environment [47]. Some Euregios have succeeded to act as a policy advisor in cross-border regions, while other multinational organizations still suffer from increased coordination costs [48].

### Subset-specific differences

From our study, we cannot say that one dimension is superior or more important than another for innovativeness because each of the dimensions can explain different levels of innovativeness. Our observation showed positive relationships between factors hampering cross-border business interaction (x-axis) and differences in innovativeness levels (y-axis) no matter which subset (i.e. new EU members, non- EU countries, and Germany) had been left out. This indicates that our results are not biased through over- or underrepresentation of specific regions. It is reasonable to assume that substitution mechanisms and overlap mechanisms in geographical and non-spatial proximity measures play a role in cross-border regions, indicating that shortcomings in one measure can be supplemented by others [49]. For example, Ferrara et al. [50] came to a similar result when they investigated the impacts of two cohesion policy interventions, i.e. in transport infrastructure and in research, technological development and innovation, in two programming periods. They concluded that both policy interventions led to the desired results, although they observed different performance outcomes [50].

We want to highlight that regional conditions promoting innovation development are not static, and it is important to ensure that framework conditions for innovativeness are constantly adapted by e.g. institutional and policy changes [see also 6, 51]. While cross-border business interaction fluctuated over time, Euregional institutions seem to have a positive impact on the level of cross-border business interaction [17]. An interplay of various stakeholders, such as enterprises, research organizations, and policy makers is important to facilitate knowledge flows across industries and hence to support innovation, i.e. a horizontal approach [52]. Additionally, bottom-up approaches should be favored over top-down approaches to foster stakeholder integration [53]. Local, Euregional authorities play an important role in fostering both approaches. Although Euregio or Euregional institutions do not explain the successful integration of border regions, they play an important role in translating ideas for economic growth [37]. Many Euregio institutions are important as a policy advisor by establishing a common forum and providing financial resources [48]. Our results can be interpreted that every region must find its own solutions [see also 54, 55].

While cooperation may be challenging in cross-border regions, these regions are also provided with opportunities which are not available for regions located further inland. Close geographical cooperation can compensate for most negative border effects, and there is still potential for increasing knowledge flows within the EU [55]. Yet, research showed that high levels of cross-border proximity did not lead to stronger cross-border economic integration [e.g. 56]. It was suggested that cross-border funding schemes such as the Interreg program offer a potential utility to support inter-regional innovation cooperation and knowledge sharing [55]. However, it is still unknown whether there is an optimal level of proximity in the dimensions of the cross-border regional innovation system providing better conditions for various stakeholders involved in cross-border cooperation which ultimately leads to an alignment of innovativeness levels in cross-border regions. Further research must be conducted to test the feasibility of fostering cross-border business interaction without decreasing differences that make collaboration interesting.

## Conclusions, implications and future outlook

Our research shows that differences in innovativeness levels within European cross-border regions can partially be explained by hampered cross-border business interaction between the countries. Our exploration of factors that define cross-border business interaction showed that obstacles in the five dimensions science and knowledge bases, socio-cultural proximity, accessibility, institutional set-up, and governance can be related to differences in innovativeness levels. European cross-border regions are diverse and the survey results were not fully representative for all European cross-border regions. Also based on our study, it was not possible to determine the relative importance of different factors in defining the level of innovativeness. However, we derive some cautious conclusions: the dimensions we investigated are interrelated, and therefore policy makers should collectively analyze them for strategic decision making. It is essential to know about specific characteristics of each region to facilitate cohesion in the EU and consequently the economy.

In practice, we suggest that a first step to increase levels of innovativeness through improvement of cross-border business interaction could be the mutual acceptance of business qualification and mutual accessibility of federal research institutions. In the short term, improving the condition of one dimension e.g. through establishing education and research facilities can increase the level of innovativeness. In the long term, all dimensions should be considered by policy makers while developing future strategies of regional development to make use of the full potential of enterprises in cross-border regions. Therefore, experts with insights into the objectives of all cross-border parties are needed in the relevant regions.

Future research should focus on two directions. First, it should concentrate on establishing coherent measures applicable at a wider level on which basic decisions can be made. Compared to non-cross-border regions, the lack of cross-border data remains a main problem of cross-border region research leading to difficulties for scientists and policy makers to estimate what effect which policy might have. A majority of websites of e.g. cross-border projects or cross-border institutions are only available in local languages, making EU wide comparisons of current policy objectives and research very difficult. A second direction for future research is the investigation of causal relations between factors hampering cross-border business interaction and levels of innovativeness. An investigation of the direct influence of dimensions on enterprises’ innovation processes is suggested to be the next research challenge for further understanding and improving the level of innovativeness in cross-border regions. From such research, we could derive suggestions about how obstacles can be overcome and even how cross-border differences can provide positive spin-offs.

## Supporting information

S1 Fig Regression results socio-cultural proximity. Specific differences of socio-cultural proximity factors: bold line = mean, continuous line = factors showing positive relationships, long dashed line = differing hierarchal structures in businesses, dashed line = different habits of addressing people, dotted line = differing approaches and attitudes in doing business (Source: own elaboration based on survey data and RIS [9]).
(TIF)

S1 Table RIS scores of respondent’s cross-border regions. Overview of the regional innovativeness in European cross-border regions participating in the survey; calculated from the indexed Regional Innovation Scoreboard **[9]**.
(PDF)

S1 File Calculation of differences in levels of innovativeness. This file explains the two-step calculation of innovativeness based on the RIS of different regions.
(PDF)

S2 File Survey paperversion. This file is the printed version of the online survey.
(PDF)
