# Supplementary figures and images for: Can differences in innovativeness between European cross-border regions be explained by factors impeding cross-border business interaction?
Source: PLoS One. 2021 Nov 11;16(11):e0258591. doi: 10.1371/journal.pone.0258591 (PMC8584766; doi:10.1371/journal.pone.0258591)

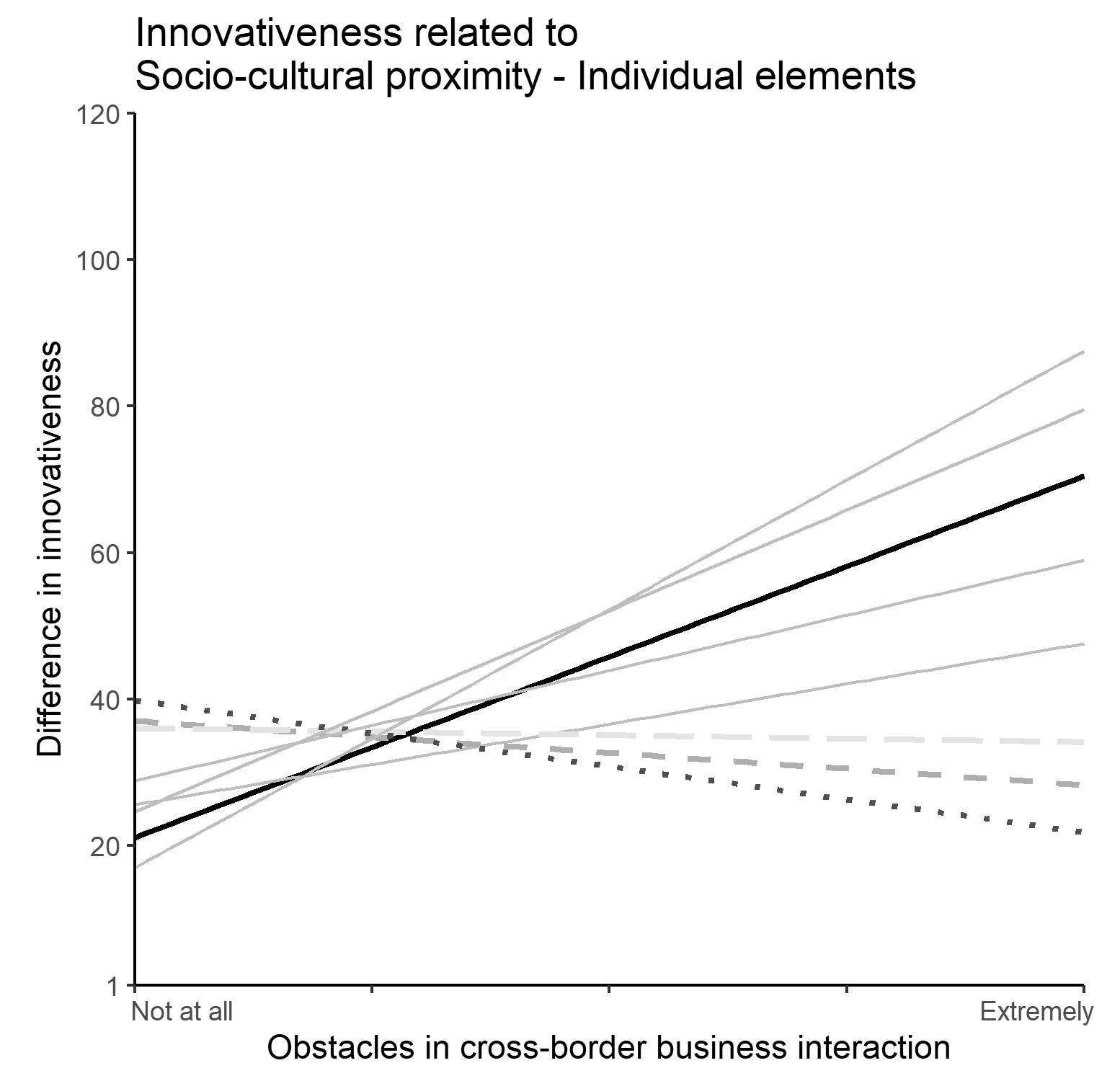

Supplement: S1 Fig — Specific differences of socio-cultural proximity factors: bold line = mean, continuous line = factors showing positive relationships, long dashed line = differing hierarchal structures in businesses, dashed line = different habits of addressing people, dotted line = differing approaches and attitudes in doing business (Source: own elaboration based on survey data and RIS [9]). (TIF) [file pone.0258591.s001.tif]
