# Supplementary material for: Can differences in innovativeness between European cross-border regions be explained by factors impeding cross-border business interaction?
Source: PLoS One. 2021 Nov 11;16(11):e0258591. doi: 10.1371/journal.pone.0258591 (PMC8584766; doi:10.1371/journal.pone.0258591)
Supplement: S1 Table — Overview of the regional innovativeness in European cross-border regions participating in the survey; calculated from the indexed Regional Innovation Scoreboard [9]. (PDF) [file pone.0258591.s002.pdf]

S1 Table. RIS scores of respondents' cross-border regions. Overview of the regional innovativeness in European cross-border regions participating in the survey; calculated from the indexed Regional Innovation Scoreboard [9]

Countries in cross-border region	Difference RIS (between countries)	Mean RIS (whole region)	"New" EU members	Non-EU countries	Germany
Norway - Sweden	6	120		x	
Finland- Sweden	7	122			
Germany - France	8	130			x
Spain – Portugal (i)	8	78			
Spain – Portugal (ii)	12	61			
Belgium- Germany -Netherlands	15	126			x
Hungary – Slovakia	16	72	x		
Germany – Netherlands (i)	18	112			x
Germany – Netherlands (ii)	19	120			x
Czech Republic – Poland	21	62	x		
Spain – Portugal (iii)	22	68			
Switzerland - France	26	139		x	
Austria - Switzerland - Germany - Liechtenstein	35	134		x	x
Czech Republic - Germany	44	81	x		x
Switzerland - Germany – France	60	140		x	x
Austria - Hungary	63	91	x		
Austria - Switzerland - Italy	82	114		x	
Germany – Denmark – Poland - Sweden-Lithuania	111	104	x		x

We received responses from different cross-border regions in Spain-Portugal and Germany-Netherlands. Hence, we calcuated the mean for the region different regions seperately and indicated this with Roman numbers (i, ii, and iii).
