# Supplementary material for: Can differences in innovativeness between European cross-border regions be explained by factors impeding cross-border business interaction?
Source: PLoS One. 2021 Nov 11;16(11):e0258591. doi: 10.1371/journal.pone.0258591 (PMC8584766; doi:10.1371/journal.pone.0258591)
Supplement: S1 File — This file explains the two-step calculation of innovativeness based on the RIS of different regions. (PDF) [file pone.0258591.s003.pdf]

Calculation of differences in levels of innovativeness

First step: A cross-border region covers an area of five NUTS regions in three countries (A, B, C), and we identified the RIS of each region (S1 File Table).

S1 File Table. Input for RIS_{diff} calculation

NUTS region	Country	RIS
1	A	80
2	A	90
3	B	100
4	C	100
5	C	110

We are interested in the innovativeness scores of the *countries* (not NUTS regions) in the cross-border region; therefore, we calculated the in-country-mean:

$$\bar{x}A = \frac{1}{2}(80 + 90) = 85$$

$$\bar{x}B = \frac{1}{1}(100) = 100$$

$$\bar{x}C = \frac{1}{2}(100 + 110) = 105$$

Second step: Among the three countries A, B, and C, we calculated the difference in innovation level (RIS_{diff}) in our example cross-border region via subtracting the highest and lowest score. Country C presented the highest score ($\bar{x}C = 105$), and country A the lowest ($\bar{x}A = 85$):

$$\text{RIS}_{\text{diff}} = \bar{x}C - \bar{x}A = 105 - 85 = 20$$

In our example, the RIS_{diff} of the cross-border region is 20 and serves as the indicator for the difference in innovativeness in the cross-border region.
