# Supplementary material for: Can differences in innovativeness between European cross-border regions be explained by factors impeding cross-border business interaction?
Source: PLoS One. 2021 Nov 11;16(11):e0258591. doi: 10.1371/journal.pone.0258591 (PMC8584766; doi:10.1371/journal.pone.0258591)
Supplement: S2 File — This file is the printed version of the online survey. (PDF) [file pone.0258591.s004.pdf]

Survey:

Cross-border Business Interaction

Dear Sir or Madam,

Within the INTERREG project "Food Pro.tec.ts" I want to investigate which factors hamper and/or stimulate and facilitate interaction between businesses located on different sides of the border. The results will be used to explore the conditions of enterprises for operating in cross-border regions. Thereby, results can help to improve the development of goals and tasks for cross-border cooperation offices in the future. Furthermore, a comparison between cross-border regions will allow to determine beneficial and unfavourable combinations of factors influencing enterprises in cross-border regions.

The completion of the questionnaire will take approximately 30 minutes. A pre-test of the questionnaire was conducted to eliminate potential misunderstandings, and to exclude questions which could not be answered immediately.
I am kindly asking you to answer all questions to the best of your knowledge and experience.
All data is processed anonymously.

Thank you very much in advance,
Sabine Neuberger

This research is conducted within the INTERREG V A-programme Germany-Netherlands and is financed by the European Regional Development Fund (ERDF) as well as the Dutch Ministry of Economic Affairs, the MWIDE NRW and the provinces Gelderland, Limburg, and Noord-Brabant. It is supervised by the project management of the Euregio Rhine-Waal.

The questionnaire will start on the next slide and is divided into the following sections:
(amount of questions can be found in brackets)

- Demographic questions (3)
- Language and customs (6)
- Infrastructure (16)
- Labour market, economic and industry structure (9)
- Role of science and knowledge bases (8)
- INTERREG projects and your cross-border cooperation organization (14)

Note:

In the following questions CB is used as abbreviation for "cross-border".

Demographic questions

First I would like to ask you some demographic questions that will be used for classification purposes.

*Please remember: In the following questions **CB** is used as abbreviation for "**cross-border**".*

1. What is your professional position in the CB cooperation organisation (Euregions)?

- ○ Director
- ○ Programme manager
- ○ Project manager
- ○ Project assistant
- ○ Other: ______

2. Which CB region do you represent or belong to?

Please write the name of your CB region!

3. Which countries belong to your CB region?

Country: ______
Country: ______
Country: ______
Country: ______
Country: ______

Language and customs

Now I would like to ask you some questions about language and customs.

4. Is a common mother tongue present in your CB region?

This question refers to the common native language(s) of your CB region, e.g. German in Germany and Austria.

- ◯ No.
- ◯ Yes, namely: ________

5. Which language(s) are commonly spoken in the CB region?

Besides the official languages of the countries, the commonly spoken language(s) can also include languages spoken by local minorities (e.g. Catalán in the French-Spanish border region, or Croatian in the Austrian-Hungarian border region).

- ☐ Basque
- ☐ Bulgarian
- ☐ Catalan
- ☐ Croatian
- ☐ Czech
- ☐ Danish
- ☐ Dutch
- ☐ English
- ☐ Estonian
- ☐ Finnish
- ☐ French
- ☐ Galician
- ☐ German
- ☐ Greek
- ☐ Hungarian
- ☐ Irish
- ☐ Italian
- ☐ Latvian
- ☐ Lithuanian
- ☐ Maltese
- ☐ Norwegian
- ☐ Polish
- ☐ Portuguese
- ☐ Romanian
- ☐ Russian
- ☐ Scottish Gaelic
- ☐ Slovak
- ☐ Slovenian
- ☐ Spanish
- ☐ Swedish
- ☐ Turkish
- ☐ Welsh
- ☐ Other: ________

6. Which other common language(s) is (are) used in business conversations in your CB region?

This question refers to languages mainly spoken in business context besides or additionally to the mother tongue (e.g. English).

7. Does there exist a common identity across the CB region, which can be expressed in "activities" taking place in either country of the CB region?

	no	yes	don't know
Annual festivities	○	○	○
Cultural events	○	○	○
Similar traditions and customs (e.g. when getting married)	○	○	○
Religious events	○	○	○
Other:	○	○	○

8. How do you assess the following statements about your CB region?

CB business interactions are hampered by ...	not at all	slightly	moderately	very	extremely	don't know
... different languages	○	○	○	○	○	○
... different habits of addressing people (e.g. greeting, first or last name)	○	○	○	○	○	○
... differing hierarchal structures in businesses	○	○	○	○	○	○
... dressing styles	○	○	○	○	○	○
... differing approaches and attitudes in doing business	○	○	○	○	○	○
... prejudice and mistrust towards businessmen from the neighbouring country	○	○	○	○	○	○
... differing working schedules (starting time & ending time of working hours, punctionality, lunch break habits)	○	○	○	○	○	○
... mistrust towards the government of the neighbouring country	○	○	○	○	○	○
... an unstable or uncertain economic situation of the neighbouring country	○	○	○	○	○	○
... differing legal systems and requirements in the neighbouring country	○	○	○	○	○	○
... differing living standards and levels of purchasing power in the neighbouring country	○	○	○	○	○	○

9. Do you know concrete examples of the last 5 years where business collaboration did not succeed due to any cultural differences?

If you also want to name and explain some examples, please use the option "Yes, namely: ...".

○ No.

○ Yes.

○ Yes, namely:
You have the option to describe the problem(s) shortly (keywords).

Infrastructure

The next section of questions focuses on infrastructural elements including transportation and information communication technology.

10. How do you assess the following statements about "natural barriers" (e.g. mountain, lakes, sea, rivers) in your CB region?

CB business interactions are hampered by ...	not at all	slightly	moderately	very	extremely	don't know
... the presence of natural barriers.	◯	◯	◯	◯	◯	◯
... longer travel distances within the CB region	◯	◯	◯	◯	◯	◯

11. Have there been attempts in the past 10 years to reduce hampering effects of "natural barriers" for CB business interactions?

- ◯ Yes.
- ◯ No.
- ◯ Don't know.
- ◯ Not applicable, because there are no hampering effects.

12. Are there plans in the next 5 years to reduce hampering effects of "natural barriers" for CB business interactions?

- ◯ Yes.
- ◯ No.
- ◯ Don't know.
- ◯ Not applicable, because there are no hampering effects.

13. Here you can describe examples of hampering effects of "natural barriers" in your CB region shortly (keywords)!

14. How do you assess the following statements about "transport infrastructure" in your CB region?

CB business interactions are hampered by ...	not at all	slightly	moderately	very	extremely	don't know	not applicable
... a lack of availability of transport infrastructure (incl. trucks, trains, and ships)	◯	◯	◯	◯	◯	◯	◯
... unequal density and quality of transport infrastructure across the CB region	◯	◯	◯	◯	◯	◯	◯
... poorly established highway infrastructure	◯	◯	◯	◯	◯	◯	◯
... poorly established train infrastructure	◯	◯	◯	◯	◯	◯	◯
... poorly established shipping infrastructure	◯	◯	◯	◯	◯	◯	◯
... poor physical condition (holes in the roads, construction works, ...) of transport infrastructure in the CB region	◯	◯	◯	◯	◯	◯	◯
... poor access to transport infrastructure (incl. trucks, trains, and ships)	◯	◯	◯	◯	◯	◯	◯

15. Have there been attempts in the past 10 years to reduce hampering effects of "transport infrastructure" for CB business interactions?

◯ Yes.
◯ No.
◯ Don't know.
◯ Not applicable, because there are no hampering effects.

16. Are there plans in the next 5 years to reduce hampering effects of "transport infrastructure" for CB business interactions?

- ○ Yes.
- ○ No.
- ○ Don't know.
- ○ Not applicable, because there are no hampering effects.

17. Here you can describe examples of hampering effects of "transport infrastructure" shortly (keywords)!

18. How do you assess the following statements about "communication infrastructure" in your CB region?

CB business interactions are hampered by ...	not at all	slightly	moderately	very	extremely	don't know
... a lack of high speed internet connection	○	○	○	○	○	○
... different usage of communication tools (e.g. rather calling than mailing, preference of paper or electronic mail)	○	○	○	○	○	○
... the costs of CB communication (e.g. higher tariffs for international calls)	○	○	○	○	○	○

19. Have there been attempts in the past 10 years to reduce hampering effects of "communication infrastructure" for CB business interactions?

- ○ Yes.
- ○ No.
- ○ Don't know.
- ○ Not applicable, because there are no hampering effects.

20. Are there plans in the next 5 years to reduce hampering effects of “communication infrastructure” for CB business interactions?

- Yes.
- No.
- Don't know.
- Not applicable, because there are no hampering effects.

21. Here you can describe examples of hampering effects of “communication infrastructure” shortly (keywords)!

22. How do you assess the following statements about “organizational infrastructure” in your CB region?

CB business interactions are hampered by ...	not at all	slightly	moderately	very	extremely	don't know
... absence of organisations facilitating interaction and cooperation	○	○	○	○	○	○
... a lack of communication across the border between institutions of the region such as agricultural chamber, economic development agencies, etc.	○	○	○	○	○	○
... a lack of available addressants for questions on the neighboring country	○	○	○	○	○	○

23. Have there been attempts in the past 10 years to reduce hampering effects of "organizational infrastructure" for CB business interactions?

- Yes.
- No.
- Don't know.
- Not applicable, because there are no hampering effects.

24. Are there plans in the next 5 years to reduce hampering effects of "organizational infrastructure" for CB business interactions?

- Yes.
- No.
- Don't know.
- Not applicable, because there are no hampering effects.

25. Here you can describe examples of hampering effects on "organizational infrastructure" shortly (keywords)!

Labor market, economic and industrial structure

The following section focuses on labor market, economic and industrial structure.

26. Are there commuters (people living in one country, but working in another) in your CB region?

Please write the names of your CB countries (one per line) and indicate whether the country sends/receives more commuters than the other countries or if it is somehow balanced.

	Unbalanced "receiving"	Unbalanced "sending"	Balanced	don't know
Country:	○	○	○	○
Country:	○	○	○	○
Country:	○	○	○	○
Country:	○	○	○	○
Country:	○	○	○	○
Country:	○	○	○	○

27. How do you assess the following statements about “economic and industrial structures” in your CB region?

CB business interactions are hampered by ...	not at all	slightly	moderately	very	extremely	don’t know
... dissimilar industrial specialization on both sides of the border	○	○	○	○	○	○
... dissimilar enterprise specific focus (e.g. high quality processed products vs. bulk production of unprocessed materials) on both sides of the border	○	○	○	○	○	○
... dissimilar enterprise demands for technical standards on both sides of the border	○	○	○	○	○	○
... differing agenda of “important issues to address” by the government is not in accordance with the agenda of the territorial member countries	○	○	○	○	○	○
... competition for qualified employees	○	○	○	○	○	○

28. Have there been attempts in the past 10 years to reduce hampering effects of “economic and industry structures” for CB business interactions?

- ○ Yes.
- ○ No.
- ○ Don’t know.
- ○ Not applicable, because there are no hampering effects.

29. Are there plans in the next 5 years to reduce hampering effects of “economic and industry structures” for CB business interactions?

- ○ Yes.
- ○ No.
- ○ Don’t know.
- ○ Not applicable, because there are no hampering effects.

30. Here you can describe examples of hampering effects of "economic and industry structures" shortly (keywords)!

31. According to your personal opinion, what is the estimated total number of enterprises located in your CB region?

Please insert a full number (no commas, dots or abbreviations).

Estimated total number:

32. According to your personal opinion, how many percent of these enterprises operate within the agri-food sector?

Please indicate the percentage (%) of agri-food enterprises – which includes every firm operating directly or indirectly within the agri-food sector – out of all enterprises within your CB region!

%

33. According to your personal opinion, how many percent of the agri-food enterprises would you consider as being innovative in your CB region?

Please indicate the percentage (%) of innovative enterprises within agri-food enterprises!

%

34. In the last 5 years, have there been activities undertaken in your CB region ....

	no	yes	don't know
... by regional/governmental/private initiatives to promote the settlement of enterprises in the CB region?	○	○	○
... by your CB cooperation organization to promote the settlement of enterprises in the CB region?	○	○	○
... by regional/governmental/private initiatives to promote innovation?	○	○	○
... by your CB cooperation organization to promote innovation?	○	○	○

Role of science and knowledge

The next section focuses on the role of science and knowledge (including education & research facilities, and research projects).

35. To your opinion and knowledge, which fields of scientific knowledge are strongly represented within the whole CB region?

Please rank the following fields of scientific knowledge starting (1) with the field of strongest representation in all areas of the CB region!

Fields		Rank
Health & Welfare	Agri-food & Forestry	1
Transport & Logistics	Engineering, Manufacturing & Construction	2
Information & Communication Technology		3
		4
		5

36. Are there differences in how enterprises access and use scientific knowledge generated and disseminated by the following institutions across the different countries in your CB region?

	no	yes	not present	don't know
Educational facilities (university, university of applied sciences)	○	○	○	○
Research facilities (incl. private research and development laboratories)	○	○	○	○
Publicly funded projects (e.g. public research and development funds)	○	○	○	○
Networking events, workshops and conferences	○	○	○	○
Research projects initiated by the EU and national/regional government (e.g. cooperation projects between educational and research facilities and industry)	○	○	○	○
Cross-border cooperation projects initiated by the EU and national/regional government	○	○	○	○

37. Here you can describe examples of the last 5 years where problems of accessing "science and knowledge institutions" located abroad occurred (keywords)!

38. To your opinion and knowledge, which of the elements are not appropriately present or available in your CB region?

Please rank the following items, starting with the most limiting/disadvantageos one!

Educational facilities	Research facilities
Research funds	Public R&D expenditure
Networking events, workshops and conferences	R&D projects (between educational/research facilities and industry)
Cross-border cooperation projects	Business partners nearby

1
2
3
4
5
6
7
8

39. Are there plans in the next 5 years to improve the situation of “science and knowledge” in your CB region?

- Yes, plans are going to be developed
- No, there is nothing planned to improve the situation.
- Don’t know.
- Not applicable, because the situation is fine.

40. Here you can describe past and future plan(s) on “science and knowledge” shortly (keywords)!

41. How do you assess the following statements about "science and knowledge" in your CB region?

**CB business interactions are hampered by ...**	not at all	slightly	moderately	very	extremely	don't know
... a lack of support by educational institutions (e.g. university, university of applied sciences)	○	○	○	○	○	○
... a lack of support by research institutions (e.g. Fraunhofer institutes, Greenport Venlo)	○	○	○	○	○	○
... a lack of projects that facilitate connections and exchange between enterprises and the research sector in the CB region	○	○	○	○	○	○
... a lack of networking events that facilitate connections and exchange between enterprises and the research sector in the CB region	○	○	○	○	○	○
... a lack of R&D funds accessible to enterprises from all countries in the CB region	○	○	○	○	○	○

42. How much money is spent per year for research and development activities in the CB region?

	< EUR 1 Mill.	EUR 1 Mill. - 5 Mill.	> EUR 5 Mill.	don't know
In total (public and private)	○	○	○	○
Through provision of research funds (by national/regional government or the EU)	○	○	○	○

INTERREG projects & your CB cooperation organization

The last section of the survey includes specific questions on Interreg projects and the role of your specific CB cooperation organization.

43. How much money was invested in total in Interreg projects in your CB region in the last 10 years?

- ◯ < EUR 1 Mill.
- ◯ EUR 1 Mill. - EUR 5 Mill.
- ◯ > EUR 5 Mill.
- ◯ Here you can enter a concrete number: ____________
- ◯ Don't know

44. Can you provide more detail on these Interreg projects, such as ...

	no	yes	don't know
Did at least one of these Interreg projects (past & current) focus on facilitating innovation?	◯	◯	◯
Before initiating Interreg projects, were the characteristics and structures of demand in your CB region analyzed?	◯	◯	◯

45. How important is innovation for Interreg projects in your CB region?

- ◯ Primary focus
- ◯ Important element
- ◯ Minor element
- ◯ Not present

46. How many stakeholders participated in Interreg projects in the past 20 years?

Please indicate the approximate amount of project partners (not people) involved!

Number of educational facilities	____________	☐ I don't know
Number of research facilities	____________	☐ I don't know
Number of enterprises	____________	☐ I don't know

47. At which percentage did stakeholders participate in SEVERAL (i.e. two or more) Interreg projects in the past 20 years?

- ◯ More than 75% of participants were the same
- ◯ About half of the participants remained the same
- ◯ Less than 25% of participants were the same
- ◯ Don't know

48. Why do you think the participant did / did not participate in such projects repeatedly?

Participation repeated, because ...	
Participation did not repeat, because ...	

49. Can you provide more information about country involvement in Interreg projects?

	very unbalanced	somewhat unbalanced	balanced weak	balanced strong	don't know
How balanced was the active participation from the neighbouring countries in the CB region?	◯	◯	◯	◯	◯
How strong is political commitment towards CB interactions at local level (e.g. community)?	◯	◯	◯	◯	◯
How strong is political commitment towards CB interactions at regional level (e.g. province, federale state)?	◯	◯	◯	◯	◯
How strong is political commitment towards CB interactions at national level?	◯	◯	◯	◯	◯

50. Can you name examples why countries were not equally involved in projects?

- ◯ Yes, I think that ...
- ◯ I don't know.
- ◯ Not applicable as political commitment in all countries was quite equal.

51. What do you consider tasks of your CB cooperation organisation?

Please choose the statement (A or B) that best describes the tasks/goals of your CB cooperation organization!

1A We want to actively establish connection between enterprises on both sides of the border	◯	◯	1B We want to assist enterprises finding potential business partners to build connections across the border.
2A We want to actively build connections between enterprises and educational research facilities in the CB region	◯	◯	2B We want to provide assistance for enterprises to build connections between them and educational research facilities in the CB region
3A We want to help the population working in the neighboring country	◯	◯	3B We want to provide information for the population working in the neighboring countries
4A We want to promote a specific industry focus	◯	◯	4B We adapt focus points to changing industry demands
5A My CB cooperation organization has an independent policy on innovation	◯	◯	5B My CB cooperation organization takes over the role of an administrative office responsible for implementing national and European policies

52. Would you please indicate, which tasks of your CB cooperation organization you consider as the most important ones?

You can name up to three, starting with most important one!

You may also use the statements from the prior question (1A to 5B).

1st priority:

2nd priority:

3rd priority:

53. Which of the following cross-border policy instruments has your CB cooperation organization been involved in facilitation and implementation within the last 10 years?

Please indicate which role your CB institution had in setting up policy instruments!

	Not present	Implemented but not involved	Involved in implementation	Initiator
A) Joint public research programs	○	○	○	○
B) Joint research infrastructure, shared access to research facilities	○	○	○	○
C) Cross-border private R&D funding programs (generic and thematic)	○	○	○	○
D) Scholarships/student exchanges	○	○	○	○
E) Joint university or other higher education programs	○	○	○	○
F) Talent attraction and retention or mobility schemes	○	○	○	○
G) Cross-border labor market measures	○	○	○	○
H) Cross-border science and technology parks	○	○	○	○
I) Cluster or network initiatives	○	○	○	○
J) Cross-border innovation advisory services (vouchers, intermediaries)	○	○	○	○
K) Advisory services to spin-off and knowledge-intensive start-ups	○	○	○	○
L) Other technology transfer centers and extension programs	○	○	○	○
M) Analytical exercises and mapping (mapping of clusters or value chains, technology foresight exercises)	○	○	○	○
N) Benchmarking and policy learning	○	○	○	○
O) Joint branding of the cross-border area	○	○	○	○

54. Pease indicate which policy instruments you consider to be the most important?

Name three, starting with most important one!
You may also use the elements from the prior question (A to O).

1st:	
2nd:	
3rd :	

55. Which other support instruments for enterprises has your CB cooperation organization been using in facilitation and implementation within the last 10 years?

Please indicate what role your CB insititution had in setting up support instruments!

	Not present	Implemented but not involved	Involved in implementation	Initiator
Personnel training	○	○	○	○
Management assistance	○	○	○	○
Tax consultancy	○	○	○	○
Information on (health) insurance	○	○	○	○
"Border information point" for all citizens on work and life in another country of the CB region	○	○	○	○
other: ____	○	○	○	○

56. Does your CB cooperation organization offer enterprises the following possibilities of interaction?

Please indicate whether the following elements are present in your CB region!

	no	yes
Meetings upon appointment or request	○	○
Meetings during regular service desk consulting hours	○	○
Other events initiated by the national government or industry	○	○
Project meetings	○	○
Others, such as: ____		

You have now reached the end of the survey.

If you have any questions, concerns or complaints about this research study, you are welcome to write them down!

Thank you for completing this questionnaire!

The researcher for this study is Sabine Neuberger who can be reached at CB-survey@hsrw.eu.
